# Apis Mellifica

**Published:** 1895-03

**Authors:** 


### HOMŒOPATHIC PHYSICIAN,

A MONTHLY JOURNAL OF

HOMŒOPATHIC MATERIA MEDICA AND CLINICAL MEDICINE.

"If our school ever gives up the strict inductive method of Hahnemann, we are lost, and deserve only to be mentioned as a caricature in the history of medicine."—CONSTANTINE HERING.

Vol. XV. MARCH, 1895. No. 3.

### EDITORIAL.

APIS MELLIFICA.—The editor has been so frequently importuned by the readers of this journal who have read the editorials on Aconite and Belladonna in the September and October numbers, to give further indications for prominent remedies taken from the notes of the venerable Dr. Lippe, that in compliance with these requests he gives the present article upon Apis.

In all his lectures Dr. Lippe never stated that any remedy upon which he was lecturing was "good" for this or that disease. He carefully abstained from such assertions lest he be accused of "putting on the pathological livery." His way of lecturing was to at once plunge into the reading of the symptoms of the particular remedy considered, and to make comments upon individual symptoms as they occurred to him, such as that it was similar to this remedy or that, or contrary as the case might be.

These comments were not only highly instructive but they were also very interesting and sometimes amusing. The indications seemed to arouse in his mind a train of associations connected with his own large experience, and he would freely relate these incidents, which, being easily remembered and withal associated with the symptoms, made an indelible impression on the student. The editor was not a writer of short-hand in those days, and so he is not able to reproduce Dr. Lippe's original style, nor to relate even a quarter part of the anecdotes which, unhappily, have faded from memory.

Imitating the plan of the great apostle of Hahnemann as well as possible after the lapse of twenty-seven years, we consider the mind and disposition of Apis.

The Apis patient is exceedingly restless and continually changing his occupation. He or she quickly tires of any occupation and of any company he may enter. He does not know what to do with himself, and feels that he has no control over his will. This makes him very unhappy. He cannot remain in the society of one set of people, but seeks the company of others. He affects hilarity while he is really very depressed.

The restlessness of Apis is otherwise peculiar, especially in bed. The patient tumbles about the bed just like a kitten. This is especially true in scarlet fever and diphtheria.

This is similar to Aconite. The reader will remember a similar statement concerning Aconite in the September editorial.

The patient is much addicted to *whining* without having any just cause, or being able to assign any reason. He "moans during sleep and whines continually while awake." This whining is similar to Hyoscyamus. The patient "whines, but knows not why." See *Guiding Symptoms*. The Muriatic Acid patient gives deep groans significant of pain. These all are Dr. Lippe's own comments.

The editor ventures to add some comparisons of his own, picked up in the course of years of practice.

This *whining* is similar to Sulphur. Sobbing, moaning, groaning can be found under Cocculus. Mercurius has constant moaning, and China has moaning and whining during sleep.

Antimonium-tartaricum has continual crying and whining of a little child for three days. It sleeps only for fifteen or twenty minutes at a time.

In reference to this symptom of whining Dr. Lippe relates the following cases:

A young man, member of a prominent Philadelphia family, was prostrated with typhoid fever. On the fourteenth day he was so ill he seemed likely to die. The chief symptom was moaning. "Not the deep, heavy moaning of Muriatic Acid," said the lecturer, "but a kind of whining without cause. This decided me to give Apis. He was relieved, and he made an excellent recovery."

"A few years later this same young gentleman was seized with small-pox. On the third day, the eruption ceased to develop and showed a disposition to disappear. At the same time the urine was suppressed. Not a drop of urine was passed for twenty-four hours. The same mental symptom cropped out as when he had the typhoid fever—constant *whining*. Nobody had ever told me to use Apis in small-pox. But there was this mental symptom. I gave Apis and the next morning the chamber was full of urine and the pustules came out as large as grapes. This was followed by an excellent recovery and *without pock marks*. Thus I confirmed this symptom in two widely different diseases. In old-school treatment when suppression of urine sets in, in small-pox such cases are hopeless. In this case not two hours had passed after taking the Apis before urine began to pass in abundance."

Dr. Lippe frequently quoted this experience with Apis years after he had ceased to lecture, and once he published it in *The Hahnemannian Monthly* from which it was copied into THE HOMŒOPATHIC PHYSICIAN.

Apis is the most important remedy in the jealousy of women; "perhaps," suggested Dr. Lippe, "because the honey bee is the most jealous creature in the world; one which will not tolerate a rival."

Apis is apt to be the remedy for delirium occurring from suppressed scarlet eruption.

The Apis patient is awkard and breaks things. He drops things. This is similar to Bovista, and Hellebore.

It is Guernsey's keynote to Hellebore.

The editor adds the following notes:

Apis; a girl generally careful, suddenly became awkward, and let things fall when handling them (Hering).

Natrum-muriaticum; drops things from nervous weakness.

Kali-carbonicum; weakness of left wrist, causing patient to drop things (David Wilson, of London).

Sepia, Cuprum, and Stannum, as well as Apis, Bovista, Hellebore, Natrum-muriaticum, and Kali-carbonicum, the remedies already mentioned, have this same symptom of awkwardness and dropping things.

The Apis patient screams out or shrieks in his sleep. This is called by the French the "Cerebral cry." It is indicative of congestion to the brain, and is common in children.

This last symptom completes the survey of the mental symptoms of Apis. It has not been the intention to write an exhaustive article, but merely to call attention to some of the most prominent mental symptoms which were commented upon by Dr. Lippe in his admirable lectures. The best idea of the mental symptoms of Apis, for those who desire more detail, can be gained from a perusal of the pathogenesis of Apis in Hering's *Guiding Symptoms.*

Next month we will speak of some other characteristics of Apis.

